# Supplementary material for: Causal Relationship between Aging and Anorexia Nervosa: A White-Matter-Microstructure-Mediated Mendelian Randomization Analysis
Source: Biomedicines. 2024 Aug 16;12(8):1874. doi: 10.3390/biomedicines12081874 (PMC11351342; doi:10.3390/biomedicines12081874)
Supplement: Supplementary file 1 [file biomedicines-12-01874-s001.zip › Supplementary Figure/Figure S4/Singe SNP plot of MR analysis for TL to ubm-b-1644.pdf]

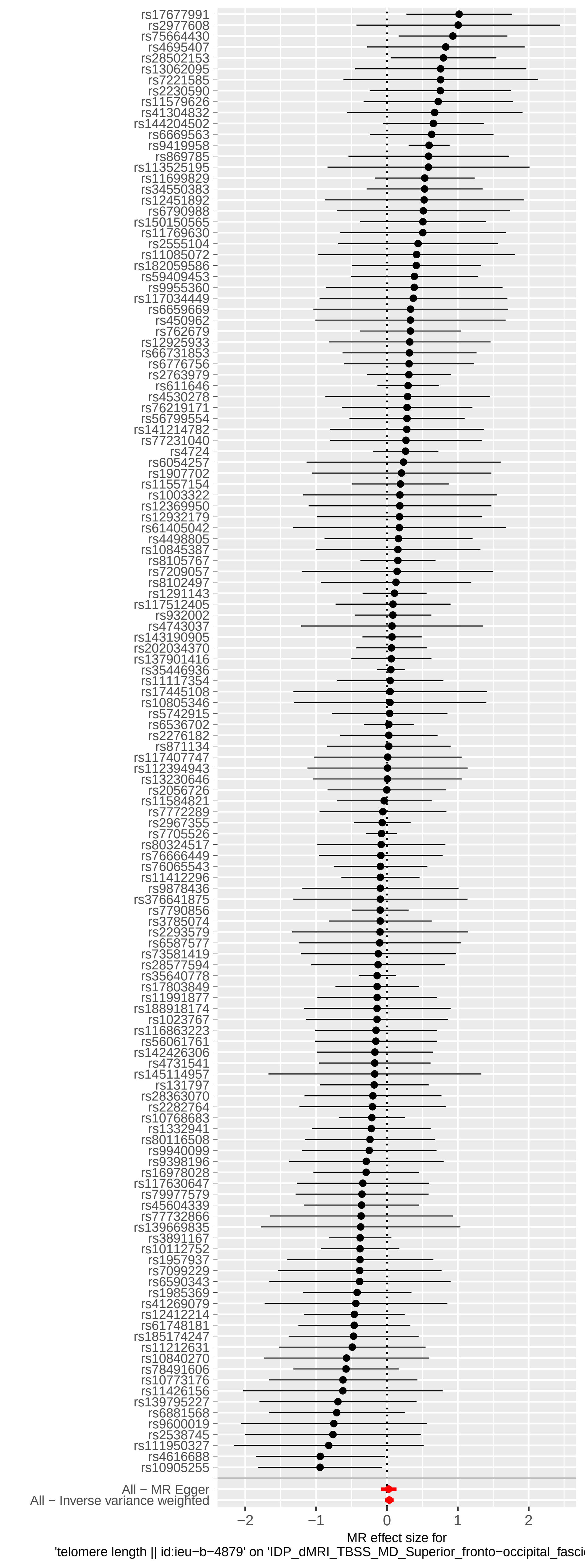

rs17677991
rs2977608
rs75664430
rs4695407
rs28502153
rs13062095
rs7221585
rs2230590
rs11579626
rs41304832
rs144204502
rs6669563
rs9419958
rs869785
rs113525195
rs11699829
rs34550383
rs12451892
rs6790988
rs150150565
rs11769630
rs2555104
rs11085072
rs182059586
rs59409453
rs9955360
rs117034449
rs6659669
rs450962
rs762679
rs12925933
rs66731853
rs6776756
rs2763979
rs611646
rs4530278
rs76219171
rs56799554
rs141214782
rs77231040
rs4724
rs6054257
rs1907702
rs11557154
rs1003322
rs12369950
rs12932179
rs61405042
rs4498805
rs10845387
rs8105767
rs7209057
rs8102497
rs1291143
rs117512405
rs932002
rs4743037
rs143190905
rs202034370
rs137901416
rs35446936
rs11117354
rs17445108
rs10805346
rs5742915
rs6536702
rs2276182
rs871134
rs117407747
rs112394943
rs13230646
rs2056726
rs11584821
rs7772289
rs2967355
rs7705526
rs80324517
rs76666449
rs76065543
rs11412296
rs9878436
rs376641875
rs7790856
rs3785074
rs2293579
rs6587577
rs73581419
rs28577594
rs35640778
rs17803849
rs11991877
rs188918174
rs1023767
rs116863223
rs56061761
rs142426306
rs4731541
rs145114957
rs131797
rs28363070
rs2282764
rs10768683
rs1332941
rs80116508
rs9940099
rs9398196
rs16978028
rs117630647
rs79977579
rs45604339
rs77732866
rs139669835
rs3891167
rs10112752
rs1957937
rs7099229
rs6590343
rs1985369
rs41269079
rs12412214
rs61748181
rs185174247
rs11212631
rs10840270
rs78491606
rs10773176
rs11426156
rs139795227
rs6881568
rs9600019
rs2538745
rs111950327
rs4616688
rs10905255
All − MR Egger
All − Inverse variance weighted
−2
−1
0
1
2
MR effect size for
'telomere length || id:ieu−b−4879' on 'IDP_dMRI_TBSS_MD_Superior_fronto−occipital_fasci
